# Ferroelectric BaTiO_3_/SrTiO_3_ multilayered thin films for room-temperature tunable microwave elements

**DOI:** 10.1186/1556-276X-8-338

**Published:** 2013-07-29

**Authors:** Ming Liu, Chunrui Ma, Gregory Collins, Jian Liu, Chonglin Chen, Andy D Alemayehu, Guru Subramanyam, Ying Ding, Jianghua Chen, Chao Dai, Yuan Lin, Melanie W Cole

**Affiliations:** 1 Electronic Materials Research Laboratory, Key Laboratory of the Ministry of Education and International Center for Dielectric Research, Xi’an Jiaotong University, Xi’an, 710049 People’s Republic of China; 2 Department of Physics and Astronomy, University of Texas at San Antonio, San Antonio, TX 78249, USA; 3 Department of Electrical and Computer Engineering, University of Dayton, Dayton, OH 45469, USA; 4 Center of High Resolution Electron Microscopy, Hunan University, Changsa, Hunan 410082, People’s Republic of China; 5 State Key Laboratory of Electronic Thin Films and Integrated Devices, University of Electronic Science and Technology of China, Chengdu, Sichuan 610054, People’s Republic of China; 6 Rodman Materials Research Directorate, U.S. Army Research Laboratory, Aberdeen Proving Ground, Aberdeen, MD 21005-5069, USA

**Keywords:** BaTiO_3_//SrTiO_3_, Multilayer, Ferroelectric thin films, Epitaxial behavior, Microwave dielectric properties

## Abstract

Ferroelectric BaTiO_3_/SrTiO_3_ with optimized *c*-axis-oriented multilayered thin films were epitaxially fabricated on (001) MgO substrates. The microstructural studies indicate that the in-plane interface relationships between the films as well as the substrate are determined to be (001)_SrTiO3_//(001)_BaTiO3_//(001)_MgO_ and [100]_SrTiO3_//[100]_BaTiO3_//[100]_MgO_. The microwave (5 to 18 GHz) dielectric measurements reveal that the multilayered thin films have excellent dielectric properties with large dielectric constant, low dielectric loss, and high dielectric tunability, which suggests that the as-grown ferroelectric multilayered thin films can be developed for room-temperature tunable microwave elements and related device applications.

## Background

Ferroelectric perovskite oxide materials have fascinated considerable attention both in scientific research and technology development due to their interesting physical properties and important application prospects in various areas such as electric, optical, and microwave devices in control systems and wireless communications. In the past two decades, the nonlinearly dielectric property of ferroelectric oxides has been utilized for various devices in tunable wireless microwave communications, such as room-temperature tunable microwave phase shifters, oscillators, filters, antennas, etc. [1-12]. Especially, ferroelectric barium strontium titanate (Ba_*x*_Sr_1−*x*_TiO_3_ or BST) thin films have been considered to be one of the most important candidates for the development of tunable microwave components. However, the relatively large dielectric insertion loss, soft mode effect, and limited figure of merit at high-frequency microwave regions still restrict practical applications in tunable microwave elements. Therefore, optimizing the microwave dielectric properties by lowering the dielectric loss tangent and enhancing dielectric tunability has become an important issue for device applications [13-19].

Multifunctional tunable ferroelectric BaTiO_3_/SrTiO_3_ (BTO/STO) heterostructures with artificial multilayer and/or superlattice structures have achieved a great enhancement on physical properties compared to the single-crystal epitaxial films of BTO, STO, and BST [20-27]. Especially, the interface and nanosize effects have been found to significantly enhance the dielectric properties from the BTO/STO multilayer system at low frequency range [28-33]. However, there are quite a few reports on high-frequency microwave properties in the gigahertz range. Recently, we have systematically studied [(BaTiO_3_)_0.4_/(SrTiO_3_)_0.6_]_*N*_ multilayered thin films and found that the high-frequency microwave dielectric properties and related physical properties can be significantly improved by optimizing the growth conditions. The optimized dielectric performance was achieved with the best value for the loss tangent (0.02) at approximately 18 GHz with each BTO layer thickness near 7.0 nm [34]. However, the high dielectric constant of near 1,600 achieved from the [(BaTiO_3_)_0.4_/(BaTiO_3_)_0.6_]_*N*_ multilayer is too high to meet the device requirements for impedance matching which is normally less than 500 [35]. To reduce the dielectric constant for meeting the impedance matching requirement, we have redesigned and further investigated a new combination of BTO/STO multilayer systems of the optimized [(BaTiO_3_)_0.5_/(BaTiO_3_)_0.5_]_16_ based on our above optimized multilayered structure. Here, we report our recent achievements on the microstructural studies and high-frequency microwave (5 to 18 GHz) dielectric measurements of [(BaTiO_3_)_0.5_/(SrTiO_3_)_0.5_]_16_ on (001) MgO substrates.

## Methods

**Figure 1:**
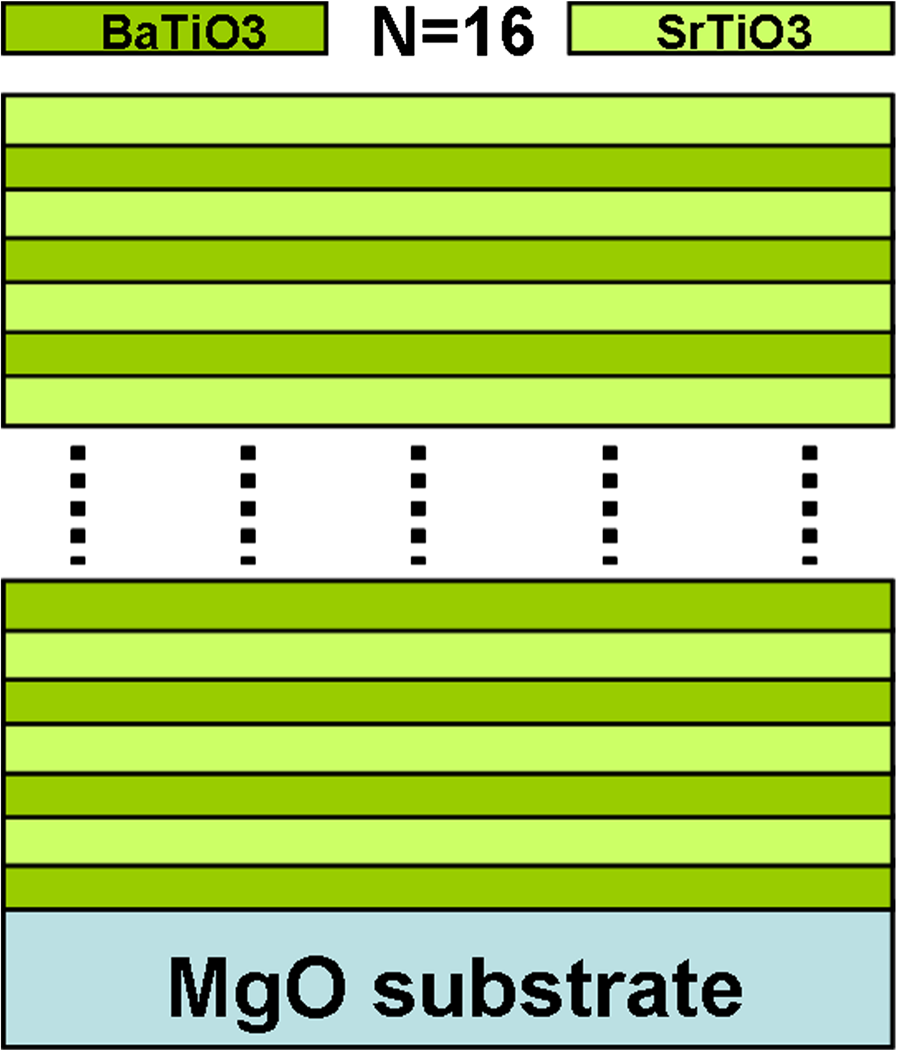
The sketch of the formula of BTO/STO superlattice structure.

A KrF excimer pulsed laser deposition system with a wavelength of 248 nm was employed to fabricate the ferroelectric BTO/STO multilayered thin films on (001) MgO substrates. Single-phase pure BTO and STO targets were employed for the fabrication. The single-crystal MgO substrates were selected for the epitaxial growth of the superlattices because of their low frequency-dependent dielectric constant (approximately 9.7) and low loss tangent values (approximately 3.3 × 10^−7^). The optimal growth conditions were found at a temperature higher than 840°C with an oxygen pressure of 250 mTorr under a laser energy density of about 2 J/cm^2^ with a repetition rate of 4 Hz. The BTO and STO layers have been designed to have the same thickness with a stacking periodic number (*N*) of 16, as seen in Figure 1. The microstructure, crystallinity, and epitaxial behavior of the as-grown multilayer were characterized by X-ray diffraction (XRD) and cross-sectional electron microscopy. The microwave dielectric properties were characterized using a coplanar waveguide (CPW) test structure consisting of an 8720C Vector Network Analyzer (Agilent Technologies, Inc., Santa Clara, CA, USA) and an on-wafer probe station. After the thru-reflect-line calibration, the swept frequency response of the *S* parameters can be obtained from the reference (CPW lines on bare MgO substrates) and test samples (CPW lines on BTO/STO multilayer-coated substrates). Details of the measurement technique can be found in the literature [36,37].

## Results and discussion

**Figure 2:**
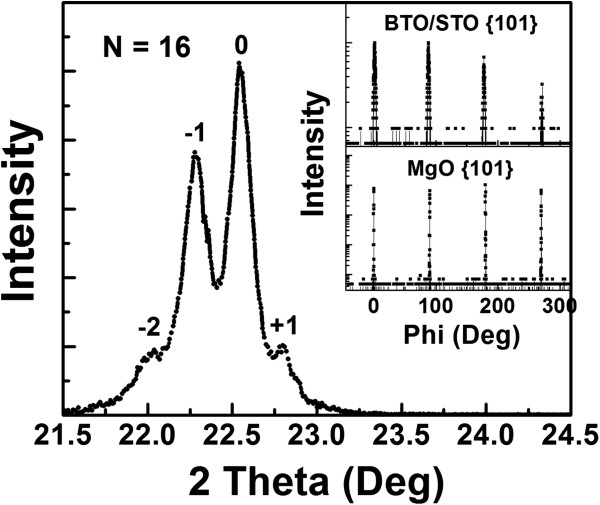
**A typical X-ray diffraction pattern of the as-grown BTO/STO superlattices on MgO substrate.** The insets are the *φ* scans taken around the {101} planes of the superlattices and MgO substrate, displaying that the films have excellent epitaxial behavior.

Figure 2 is the typical XRD pattern of the as-grown [(BaTiO_3_)_0.5_/(SrTiO_3_)_0.5_]_16_ multilayered thin films on the (001) MgO substrate with a total thickness about 500 nm. Only (00 *l*) peaks appear in the *θ*-2*θ* scans for the multilayer and substrate, indicating that the multilayer is *c*-axis oriented or perpendicular to the substrate surfaces. The rocking curve measurements from the (002) reflection of the multilayer show that the full width at half maximum is about 0.9°, indicating that it has good single crystallinity and epitaxial quality. However, three additional peaks at 2*θ* ≈ 22.04, 2*θ* ≈ 22.28, and 2*θ* ≈ 22.79 appeared, which were identified as the satellite peaks of the (002) reflection. Thus, the multilayer thickness can be estimated from these satellite peaks using the standard formula *L* = [*λ*_Cu(*K*α)_/(sin*θ*_*n* + 1_ − sin*θ*_*n*_)] [38], where *λ*_Cu(*K*α)_ is the wavelength of the Cu(*K*α) radiation and *n* corresponds to the *n*th satellite peak. Therefore, the thickness of every periodic layer (*L*) was found to be about 35 nm, giving the overall multilayer thickness of about 560 nm. This result is in good agreement with the multilayer design. The *ϕ* scans were also employed to study the epitaxial quality and the in-plane relationships between the multilayer and the substrate. The insets of Figure 2 are the *ϕ* scans taken from the {101} planes of the superlattices and MgO substrate. Only fourfold symmetric {101} reflections with sharp peaks were presented in the scans, suggesting that the multilayer has good single crystallinity and epitaxial quality. The in-plane interface relationships between the multilayer and the MgO substrate are therefore determined to be [100]_STO_//[100]_BTO_//[100]_MgO_ and (001)_STO_//(001)_BTO_//(001)_MgO_. These interface relationships indicate that the multilayer has the cube-on-cube epitaxial growth nature.

**Figure 3:**
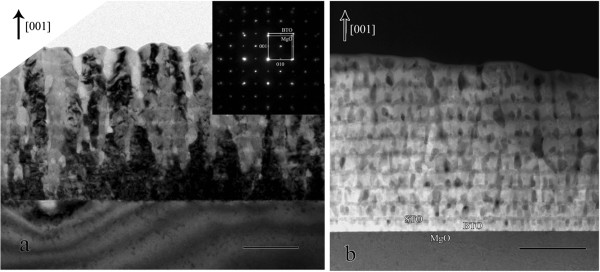
**Cross-sectional bright-field and high-angle annular dark-field image of BTO/STO superlattice thin film. (a)** Bright-field image. **(b)** HAADF-STEM image. Bar = 200 nm.

To further understand the epitaxial nature and interface structures of the as-grown multilayer, high-resolution transmission electron microscopy (HRTEM) and high-angle annular dark-field scanning transmission electron microscopy (HAADF-STEM) were employed to study the interface microstructure of the BTO/STO multilayer on (001) MgO substrates. Figure 3a is a bright-field TEM image of the ferroelectric BTO/STO multilayer grown on the (001) MgO substrate. The multilayered structures can be clearly seen from HRTEM images. The inset is a selected area electron diffraction pattern taken at the film/substrate interface with the electron beam direction parallel to the [100]_MgO_. The interface relationship of the as-grown BTO/STO multilayer was determined to be (001)_BTO/STO_//(001)_MgO_ and [100]_BTO/STO_//[100]_MgO_ with respect to the MgO substrate. Figure 3b is the HAADF-STEM image showing the multilayered structure with sharp interface structures. The electron diffraction, HRTEM, and HAADF-STEM studies on the as-grown multilayer suggest that the films have good single crystallinity and epitaxial quality.

**Figure 4:**
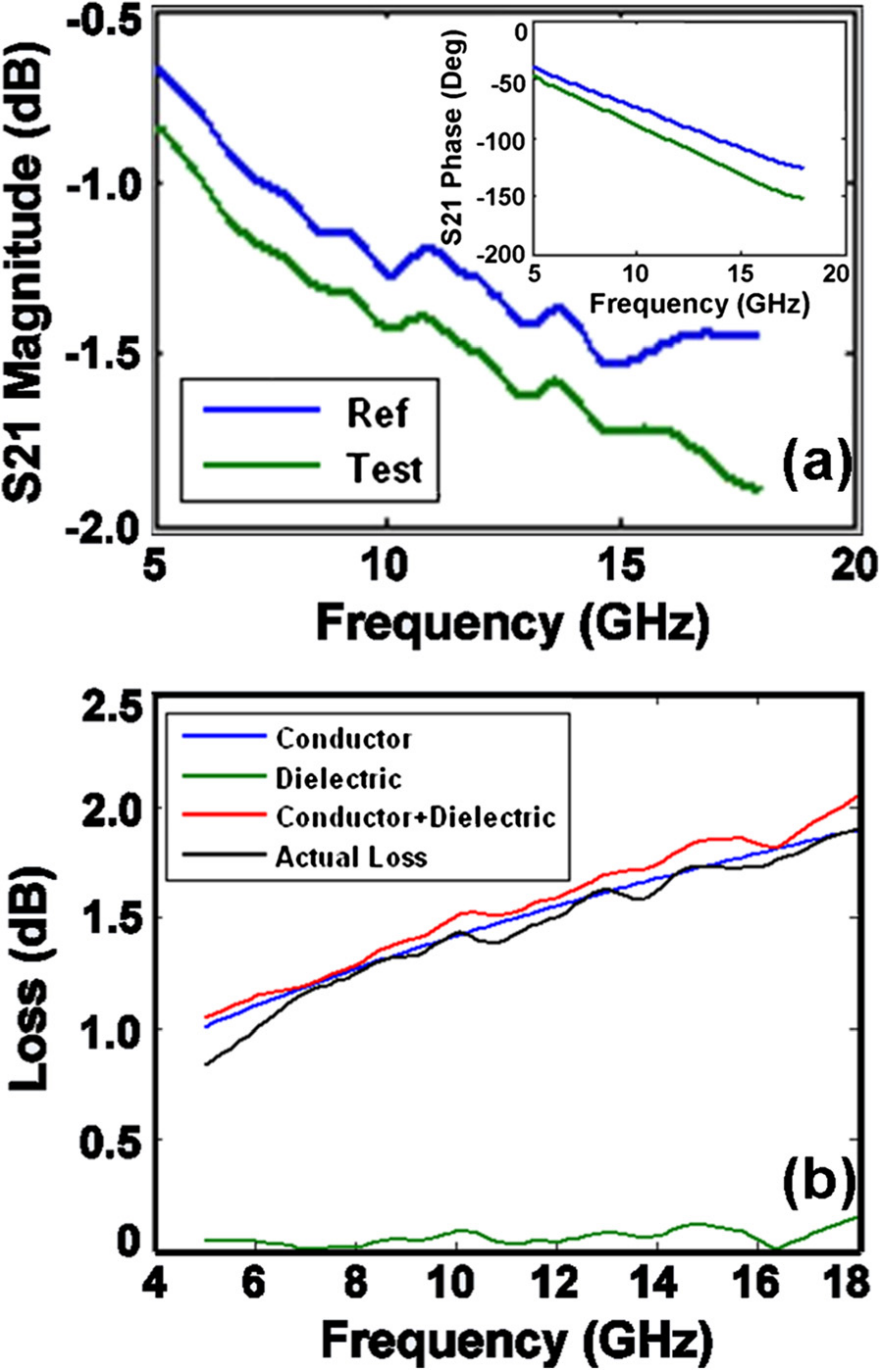
**Plots of (a) insertion loss and (b) calculated and measured conductor loss and dielectric loss** The inset in **(a)** is the relative insertion phase of *S*_21_.

The CPW test structure was used to determine the high-frequency microwave dielectric properties of the BTO/STO superlattices on (001) MgO. The test structures were fabricated on the bare MgO substrate (reference sample or ‘Ref’) and the multilayer (test sample or ‘Test’) to determine the attenuation and phase constants with and without the film test samples, which were used to compare the propagation characteristics between the reference and test samples. Figure 4a shows the swept frequency responses for the reference and test samples from 5 to 18 GHz. It can be seen that the insertion loss contribution from the multilayer is only about approximately 0.17 dB at 5 GHz and approximately 0.45 dB at 18 GHz, indicating that the films have low insertion loss at these frequencies. The inset of Figure 4a is the plot of the relative insertion phase of *S*_21_ for the reference and test samples. The total relative phase of S21 in degrees can be obtained by adjusting the phase of *S*_21_ to a lagging phase. From the magnitude and the relative phase of *S*_21_, we can obtain the attenuation and phase constant for the reference and test samples. Figure 4b shows the calculated and the measured conductor loss and dielectric loss in the sample. It is clearly seen that the calculated and measured total losses are well matched.

**Figure 5:**
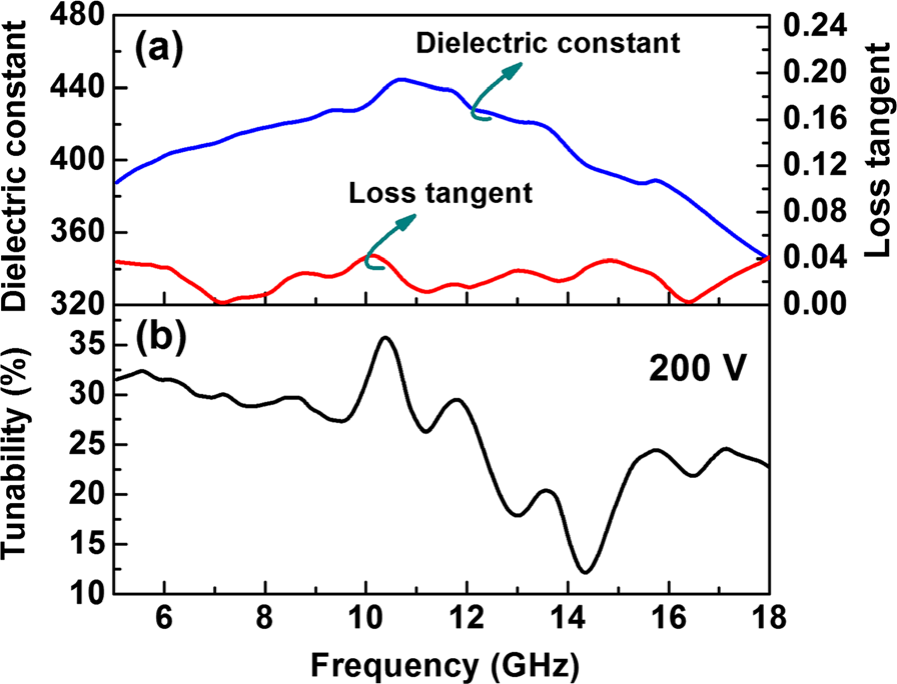
Plots of (a) relative dielectric constant and loss tangent and (b) tunability of BTO/STO superlattices.

Once the capacitance of the films was determined, the relative dielectric constant of the film *ϵ*_film_ can be obtained by conformal mapping technique [33], *C*_film_ ≅ *ϵ*_o_ (*ϵ*_film_ − *ϵ*_sub_) 2 *t*/*s*, if the limit of film thickness *t* « *s*, where *s* is the spacing distance between the center conductor and the ground line for the CPW line, and *ϵ*_sub_ is the relative dielectric constant of the substrate. Figure 5a shows the frequency dependence of the relative dielectric constant and the loss tangent for the multilayer. The relative dielectric constant and the loss tangent are varying from 340 to 445 and from 0.001 to 0.04, respectively. A maximum dielectric constant of approximately 445 at 10.65 GHz and a minimum dielectric loss of approximately 0.001 at 7.15 and 16.425 GHz were found. Figure 5b is the plot of the tunability versus the frequency of the multilayer, showing that a large dielectric tunability of 12% to 35% has been achieved from 5 to 18 GHz with a bias voltage of 200 V or an applied field of 200 kV/cm. These results indicate that the optimized dielectric performance for such a designed multilayer occurs at 10 to 12 GHz with an optimized dielectric constant of 445, a dielectric loss of 0.01, and a dielectric tenability of 35%. Overall, the microwave dielectric property of the BTO/STO multilayer on (001) MgO suggests that this system can be developed for room-temperature tunable microwave elements and related device applications.

## Conclusions

In summary, ferroelectric BTO/STO multilayers have been epitaxially grown on (001) MgO by pulsed laser deposition. The microstructural studies from X-ray diffraction show that the as-designed multilayers are *c*-axis oriented with good epitaxial nature. The high-frequency microwave (5 to 18 GHz) dielectric measurements reveal that the multilayers have excellent microwave dielectric properties with very low dielectric loss and high dielectric tenability, which suggests that the BTO/STO multilayers on (001) MgO have great potential for the development of room-temperature tunable microwave elements and related applications.

## Competing interests

The authors declare that they have no competing interests.

## Authors’ contributions

GC and CC designed and set up the experimental system. ML and CC planned the experiments. ML fabricated the films with the assistance of CM, GC, and JL. ADA and GS conducted the measurement of high-frequency microwave dielectric properties. YD and JC performed the electron microscopy studies. CD and YL performed the X-ray diffraction characterizations. MWC assisted in the data analysis. ML and CC wrote the manuscript. All authors read and approved the final manuscript.
